# Conversion Therapy of Intrahepatic Cholangiocarcinoma Is Associated with Improved Prognosis and Verified by a Case of Patient-Derived Organoid

**DOI:** 10.3390/cancers13051179

**Published:** 2021-03-09

**Authors:** Zhiwei Wang, Yun Jin, Yinghao Guo, Zhenhua Tan, Xiaoxiao Zhang, Dan Ye, Yuanquan Yu, Shuyou Peng, Lei Zheng, Jiangtao Li

**Affiliations:** 1 Department of Surgery, Second Affiliated Hospital, Zhejiang University School of Medicine, Hangzhou 310009, China; wangzw824@zju.edu.cn (Z.W.); 2515045@zju.edu.cn (Y.J.); guoyinghao07@zju.edu.cn (Y.G.); tanzh99@163.com (Z.T.); zxiaoxiao204@163.com (X.Z.); 2316030@zju.edu.cn (D.Y.); zjuyyq@zju.edu.cn (Y.Y.); zrwkpsy@zju.edu.cn (S.P.); 2 Department of Oncology, Sidney Kimmel Cancer Center at Johns Hopkins University School of Medicine, Baltimore, MD 21231, USA

**Keywords:** intrahepatic cholangiocarcinoma, conversion therapy, organoid, overall survival, genetic profiles, drug screening

## Abstract

**Simple Summary:**

The first core tip of this study is that we summarized the experience of conversion therapy of intrahepatic cholangiocarcinoma (IHCC) in a single center and found conversion therapy could provide a significant improvement in the overall survival of patients with IHCC. Previous studies mainly focused on case reports and case series, and our research provided more evidence for the efficacy of conversion therapy in IHCC. The second is that we established the organoid of IHCC for drug screening and firstly verified the results of drug screening tests in cancer organoid were consistent with the levels of efficacy observed in the patient from whom it was derived. Cancer organoid is a promising technology for conversion therapy according to our study but more organoids are still needed.

**Abstract:**

This study was performed to determine the efficacy of conversion therapy in intrahepatic cholangiocarcinoma (IHCC) and explore the feasibility of cancer organoid to direct the conversion therapy of IHCC. Patient data were retrospectively reviewed in this study and cancer organoids were established using tissues obtained from two patients. A total of 42 patients with IHCC received conversion therapy, 9 of whom were downstaged successfully, and another 157 patients were initially resectable. Kaplan–Meier curves showed that the successfully downstaged patients had a significantly improved overall survival compared to those in whom downstaging was unsuccessful (*p* = 0.017), and had a similar overall survival to that of initially resectable patients (*p* = 0.965). The IHCC organoid was successfully established from one of two obtained tissues. Routine hematoxylin and eosin staining and immunohistological staining found the organoid retained the histopathological characteristics of the original tissues. Whole exome sequencing results indicated the IHCC organoid retained appropriately 87% of the variants in the original tissue. Gemcitabine and paclitaxel exhibited the strongest inhibitory effects on the cancer organoid as determined using drug screening tests, consistent with the levels of efficacy observed in the patient from whom it was derived. This study indicates that conversion therapy could improve the survival of patients with IHCC despite its low success rate, and it may be directed by cancer organoids though this is merely a proof of feasibility.

## 1. Introduction

Intrahepatic cholangiocarcinoma (IHCC) is a rare and highly aggressive epithelial cancer accounting for 10–15% of all primary malignant liver tumors [1]. The incidence of IHCC has increased during the past two decades and shows a rise in the rate of mortality [2]. To date, surgical resection is the only potentially effective treatment for IHCC. However, the recurrence rates after resection have been reported to range from 40–80% [3]. Moreover, most patients with IHCC are diagnosed at an advanced stage owing to the lack of specific symptoms, rendering them unsuitable for surgical resection [4]. In patients with unresectable IHCC, conventional chemotherapy and radiotherapy are possible treatment options; however, these techniques are met with limited efficacy. Generally, the five-year survival of patients with IHCC is less than 5% [5,6].

To improve overall survival, several novel approaches have been proposed in recent decades. The concept of conversion therapy was proposed by Bismuth H. et al. in 1996, which demonstrated that initially unresectable malignant tumors can be downstaged using systemic or local treatment, such that patients could likely undergo surgical resection [7]. This technique was first used in the treatment of colorectal cancers (CRC) and is currently used widely in the treatment of various cancers including gastric and pancreatic cancers (PC) and hepatocellular carcinoma (HCC) [8,9,10]. Recently, the efficacy of conversion therapy in patients with IHCC was verified using several studies, which indicated that adequate tumor downstaging was achieved by systemic or local treatment and patients could obtain a relatively satisfactory prognosis [11,12,13,14,15,16,17]. However, most of these studies are case reports or case series containing no more than 10 patients. Thus, the overall success rate of conversion therapy is still unclear. Edeline J. et al. reported that only 22% of patients with locally advanced IHCC receiving radioembolization plus chemotherapy could be successfully downstaged to surgical resection owing to the low response rate of IHCC [18]. Therefore, the efficacy of conversion therapy in IHCC needs to be further improved.

Appropriate in vitro models of cancer have been explored for over 100 years. Conventional two-dimensional (2D) culture systems force cancer cells to adhere to an artificial substrate, which restricts the formation of a multi-dimensional structure. The formation of a monolayer morphology and the lack of contact with other normal cells makes it nearly impossible to engineer cancer cells to mimic the important characteristics of cancer tissues in vivo [19]. A novel three-dimensional (3D) culture system, also called an organoid, has been developed in recent times to address the defects of a 2D culture system. Compared to the 2D culture system, organoid technology provides a suitable extracellular matrix as a scaffold for the attachment of cells to form a multi-dimensional structure [20]. Consequently, cancer organoids can mimic the structure and function of original cancer tissues, including CRC, PC, and HCC [21,22,23]. High-throughput or medium-throughput drug screening of original cancer tissues is possible using organoids [24]. However, organoids of IHCC have been established, evaluated, and reported only in a few studies [23]. There remain uncertainties regarding whether a cancer organoid can be successfully used to determine and direct the anti-tumor treatment of IHCC, especially conversion therapy.

This study was undertaken to determine the efficacy of conversion therapy in patients with IHCC based on a retrospective review. Moreover, a cancer organoid was established to determine the feasibility of directing the conversion therapy of IHCC.

## 2. Materials and Methods

### 2.1. Patient Selection

Patients who were diagnosed with IHCC between January 2013 and June 2020, in the Second Affiliated Hospital, Zhejiang University, School of Medicine, were retrospectively reviewed. The following patients were excluded from this study: (1) those with remote lymph node metastases, large vessel invasion, or diffuse multiple lesions, (2) those not receiving any specific anti-tumor treatment, (3) those suffering from several types of cancers, and (4) those who had received anti-tumor treatment at other medical centers. This study was performed in compliance with the principles of the Declaration of Helsinki and was approved by the ethics committee of the Second Affiliated Hospital, Zhejiang University, School of Medicine (No. 2019-408). Informed consent from the included patients was not obtained owing to the retrospective nature of this study.

### 2.2. Data Collection

To analyze the relationship between treatment patterns and prognosis in patients with IHCC, the demographics (age and gender), details on liver cirrhosis, number of lesions, tumor size, vascular invasion, perineural invasion, histological grade, AJCC TNM stage, residual tumor after surgical resection, perioperative adjuvant treatment, systemic treatment, and imaging examination date of the included patients were collected at admission. Patients were divided into initially resectable group, successfully downstaged group, and unsuccessfully downstaged group. All data were collected by two independent investigators and conflicts, if any, were resolved through consultation.

### 2.3. Follow-Up

Considering the relatively short survival time of patients with IHCC, all included patients were followed up for at least three months. The recurrence and metastasis of IHCC were recorded during follow up.

### 2.4. Organoid Establishment

Two tissue samples of resected IHCC were collected from two independent patients whose tumors were downstaged successfully after conversion therapy. The tissues were used to establish patient-derived cancer organoids to determine their effectiveness in directing the treatment pattern in conversion therapy. The experimental procedures were approved by the ethics committee of the Second Affiliated Hospital, Zhejiang University, School of Medicine, and informed consent from patients was obtained before tissue collection.

The obtained tissues were shipped on ice within 4 h. The tumor tissues were cut into small pieces (appropriately 1–2 mm^3^) and incubated in 2 mg/mL of collagenase D (Roche, Basel, Switzerland) for 2 h at 37 °C. The isolated cells were passed through a 100-µM cell strainer (Corning, Basel, Switzerland), pelleted using centrifugation at 300× *g* for 5 min at room temperature, and embedded in a mixture of Matrigel matrix (growth factor reduced; Corning, Corning, NY, USA) and culture medium on ice in a 1:1 ratio.

Appropriately 2−5 × 10^5^ cells in 100 μL of Matrigel matrix mixture were seeded into 24-well plates (Corning, Corning, NY, USA) after the plates were coated using 200 μL of the Matrigel matrix mixture. Then, cells were overlaid with 500 µL of culture medium. The culture medium contained advanced Dulbecco’s modified Eagle medium/F12 (Gibco, Carlsbad, CA, USA) supplemented with 1× penicillin/streptomycin (ThermoFisher, Waltham, MA, USA), 1× Glutamax (ThermoFisher, Waltham, MA, USA), 10 mM N-2-Hydroxyethylpiperazine-N-2-Ethane Sulfonic Acid (ThermoFisher, Waltham, MA, USA), 1× B27 supplement (Gibco, Carlsbad, CA, USA), 1× N2 supplement (Gibco, Carlsbad, CA, USA), 10 mM nicotinamide (Sigma, St. Louis, MO, USA), 1.25 mM N-acetyl-L-cysteine (Sigma, St. Louis, MO, USA), 10 nM gastrin (Sigma, St. Louis, MO, USA), 5 μM A83-01 (Tocris, Bristol, UK), 50 ng/mL recombinant human epidermal growth factor (PeproTech, Rocky Hill, NJ, USA), 100 ng/mL recombinant human fibroblast growth factor 10 (PeproTech, Rocky Hill, NJ, USA), 10 µM Y-27632 (Tocris, Bristol, UK), 500 ng/mL recombinant human R-Spondin1 (PeproTech, Rocky Hill, NJ, USA), 10% Afamin/Wnt3a CM (MBL Life Science, Kyoto, Japan), and 100 ng/mL recombinant human Noggin (PeproTech, Rocky Hill, NJ, USA). The culture medium was replaced every 4–5 days.

### 2.5. Preparation of Histological Sections

Primary tissue samples were fixed in 10% neutral-buffered formalin and embedded in paraffin blocks using a standard procedure. Organoids were released from the Matrigel Matrix by incubating them in the Cell Recovery Solution (Corning, Corning, NY, USA) for 1 h, according to the manufacturer’s instructions. Organoids were centrifuged at 300× *g* for 5 min at room temperature and fixed in 10% neutral-buffered formalin for 2 h. Next, the organoids were embedded into 4% low-melting agarose after centrifugation, and the agarose blocks were embedded into paraffin. Sections of primary tissues and organoids (4-μM thick) were prepared and subjected to routine hematoxylin and eosin (H&E) staining and immunohistological staining. For immunohistological staining, mouse monoclonal antibodies against cytokeratin-7 (CK7, Abcam, Cambridge, UK, 1:2000) and epithelial cell adhesion molecule (EpCAM, Abcam, Cambridge, UK, 1:500) were used.

### 2.6. Whole Exome Sequencing (WES)

Genomic DNA of primary tissues and organoids were extracted using GenElute Mammalian Genomic DNA miniprep kits (Sigma, St. Louis, MO, USA). The quality of the extracted genomic DNA was verified by monitoring DNA degradation using 1% agarose gels and measuring the DNA concentration using Qubit DNA Assay kit (Invitrogen, Carlsbad, CA, USA). The exome sequences were enriched from 0.4 μg of genomic DNA using an Agilent liquid capture system (Agilent SureSelect Human All Exon V6) according to the manufacturer’s guidelines. Clustering was performed using a cBot Cluster Generation System and an Illumina PE Cluster kit (Illumina, San Diego, CA, USA) according to the manufacturer’s guidelines. Sequencing was performed using an Illumina platform and 150 bp paired-end reads were generated.

### 2.7. Drug Treatment

The cancer organoids were released from the Matrigel matrix by incubating in the Cell Recovery Solution (Corning, Corning, NY, USA) for 1 h followed by the incubation in 0.25% trypsin-ethylene diamine tetraacetic acid solution to allow its dissociation into single cells. These single cells were resuspended in Matrigel Matrix mixture on ice and approximately 500–1000 cells were embedded in a 384-well plate. The cells were left undisturbed to recover for 2 days. Then, the culture medium was replaced with fresh medium containing 0.01, 0.1, 1, 10, or 50 µM of gemcitabine, 5-fluorouracil (5-FU), cisplatin, paclitaxel, infigratinib, or ivosidenib. After incubation with different drugs for 72 h, the viability of cancer cells was determined using a CellTiter-Glo 3D Cell-Viability assay (Promega, Madison, WI, USA). The viability of cancer cells incubated in a culture medium with phosphate-buffered saline served as a negative control. The screening of each drug was performed in triplicate.

### 2.8. Statistical Analysis

Continuous variables in this study are expressed as medians with interquartile ranges and compared using the Mann–Whitney U test. Categorical variables are expressed as a number using percentages and compared using the Chi-squared test. The Kaplan–Meier method and log-rank test were used to compare differences in overall survival among successfully downstaged, unsuccessfully downstaged, and initially resectable patients. SPSS Statistics 22.0 (IBM Corporation, New York, NY, USA) was used for statistical analysis and a two-tailed *p*-value of <0.05 was considered statistically significant.

## 3. Results

### 3.1. Patient Characteristics

**Figure 1:**
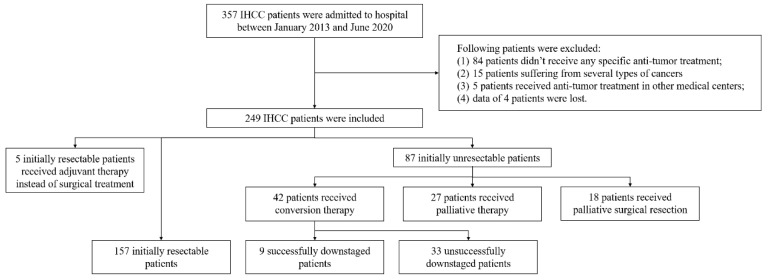
Flow chart of this study.

Patients with IHCC admitted to our hospital between January 2013 and June 2020 were retrospectively reviewed as depicted in the flow chart (Figure 1). A total of 357 patients were diagnosed with IHCC, of which 108 were excluded from this study based on the exclusion criteria. There were 162 initially resectable and 87 initially unresectable patients among the remaining 249 patients. Forty-five initially unresectable patients had distant metastasis at the time of diagnosis, 27 and 18 of whom received palliative adjuvant therapy and palliative surgical resection, respectively. The remaining 42 of the 87 patients received conversion therapy, of which 9 (21.4%) were downstaged successfully to further receive curative surgical resection.

The data of patients receiving conversion therapy are listed in Table 1. No significant differences were found with respect to age, gender, liver cirrhosis, multiple lesions, vascular invasion, and perineural invasion between successfully downstaged and unsuccessfully downstaged patients. Most tumors of enrolled patients were poorly differentiated; however, histological grades of 20 unsuccessfully downstaged patients (60.6%) were unknown owing to insufficient diagnosis after obtaining biopsy samples using fine-needle aspiration. Moreover, there were several T2-stage tumors in the unsuccessfully downstaged group (23 patients, 69.7%) than in the successfully downstaged group (2 patients, 22.2%).

### 3.2. Survival Analysis

The detailed characteristics of patients who were downstaged successfully are presented in Table 2. Transcatheter arterial chemoembolization was used as the treatment protocol of conversion therapy in two patients. One of the patients received radiotherapy for the tumor surrounding the large vessels. The remaining six patients received gemcitabine-based chemotherapy to downstage the tumor, whereas one patient received toripalimab in addition to gemcitabine-based chemotherapy.

On the other hand, detailed conversion strategies in 33 unsuccessfully downstaged patients were as follows: 13 patients receiving gemcitabine-based chemotherapy, 5 patients receiving transcatheter arterial chemoembolization, 4 patients receiving radiofrequency ablation, 4 patients receiving 5-fluorouracil monotherapy, 2 patients receiving radiotherapy, 2 patients receiving immunotherapy, 1 patient receiving targeted therapy, and 2 patients receiving immunotherapy combined with targeted therapy.

**Figure 2:**
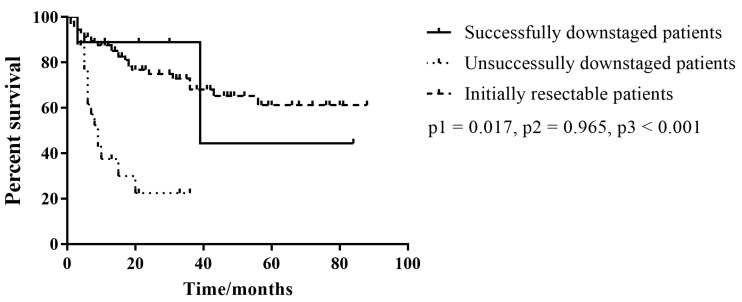
Kaplan–Meier curves comparing overall survival of successfully downstaged patients, unsuccessfully downstaged patients, and initially resectable patients based on the log-rank test. p1: successfully downstaged patients versus unsuccessfully downstaged patients; p2: successfully downstaged patients versus initially resectable patients; p3: unsuccessfully downstaged patients versus initially resectable patients.

All patients were followed up for at least 3 months. Two patients succumbed during the follow-up period, one of whom succumbed 39 months after diagnosis owing to lung metastasis of cancer, while the other succumbed to postoperative complications. An overall survival curve was generated based on the Kaplan–Meier method and log-rank test as shown in Figure 2. There was a significant improvement in the overall survival of successfully downstaged patients compared to the unsuccessfully downstaged patients (*p* = 0.017). Moreover, successfully downstaged patients had an overall survival similar to that of initially resectable patients (*p* = 0.965).

### 3.3. Organoid Establishment

**Figure 3:**
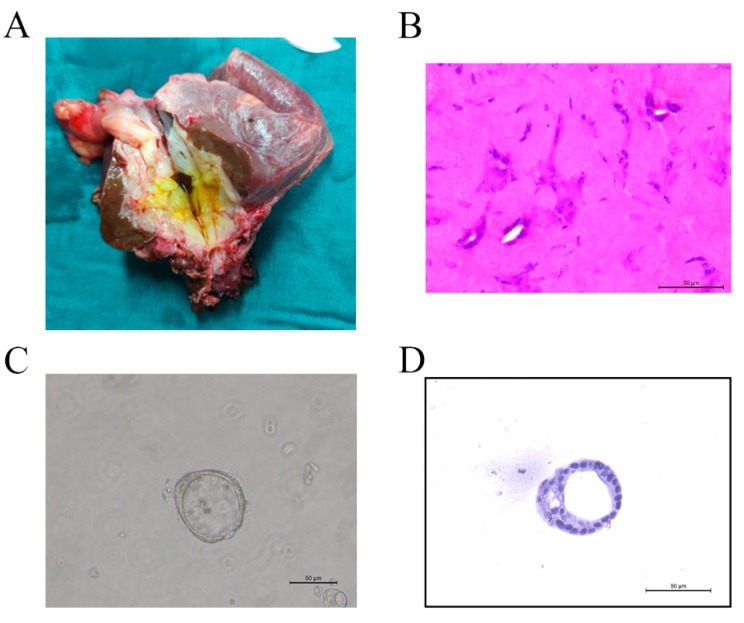
Histopathological characterization of cancer organoid. (**A**) Gross specimen and (**B**) H&E staining of intrahepatic cholangiocarcinoma (IHCC) tissue, and (**C**) bright-field image and (**D**) H&E staining of the cancer organoid. Scale bar, 50 μM.

**Figure 4:**
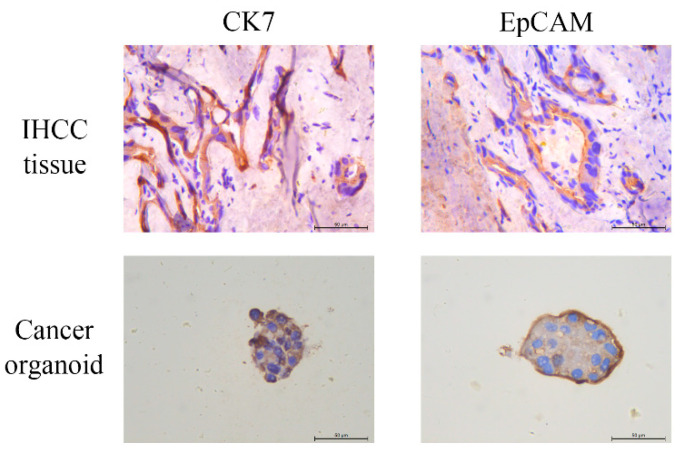
Immunohistological staining of IHCC tissue and cancer organoid, including CK7 and EpCAM. Scale bar, 50 μM.

To determine the effectiveness of cancer organoids in developing a treatment pattern in IHCC conversion therapy, a cancer tissue was collected from patient 8 and patient 9 to establish cancer organoids. However, one of the cancer organoids from patient 9 was not established successfully owing to the extent of necrosis in the collected sample. Gross specimen and H&E staining results of the other IHCC tissue are shown in Figure 3A,B. There were remnant nests of malignant cells in the obtained IHCC tissue owing to effective conversion therapy. Cancer organoid of this IHCC tissue was successfully established and was found to exhibit a monolayered cystic structure as seen in the bright field image (Figure 3C). H&E staining of the cancer organoid (Figure 3D) suggested that it could mimic the structure and function of the original IHCC tissues. Some specific tumoral markers were tested by immunohistological staining, such as CK7 and EpCAM, as shown in Figure 4. It was found that expression profiles of cancer organoid resembled original tissue. CK7 and EpCAM were highly expressed in both organoid and tissue.

**Figure 5:**
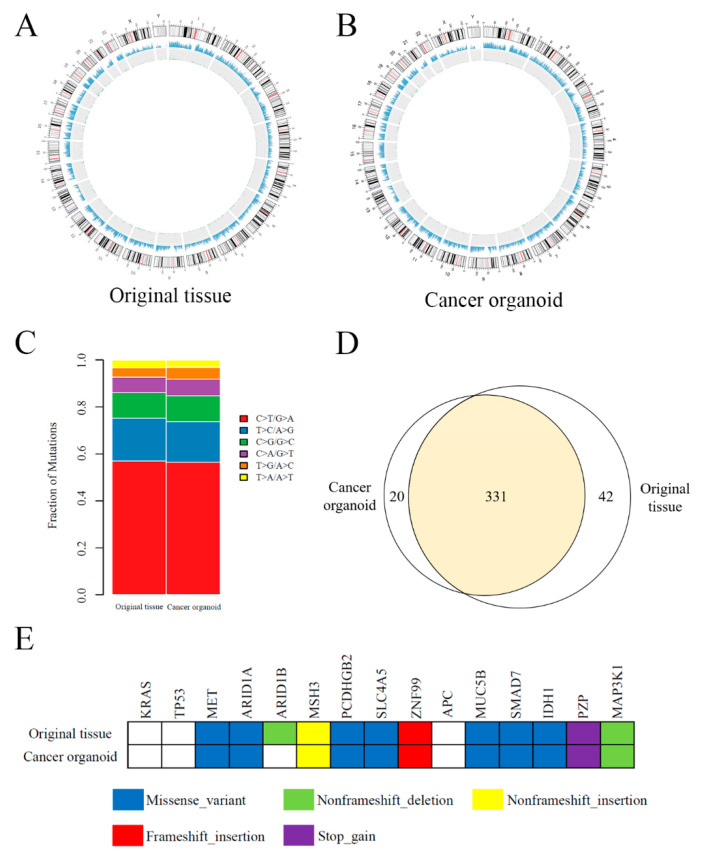
Whole exome sequencing of original tissue and the cancer organoid. (**A**,**B**) Circos plots of the original tissue and cancer organoid. (**C**) The distribution of base substitutions in the organoid and original tissue. (**D**) Venn diagram demonstrating 89% overlap of single nucleotide variants between the original tissue and cancer organoid. (**E**) Representative variants in the original tissue and cancer organoid.

WES was performed by extracting the total DNA of the cancer organoid and original tissue to determine if the organoid retained the gene expression profiles of the original tissue. The circos plots of the original tissues and cancer organoids depicted in Figure 5A,B show a similar distribution. The distribution of base substitutions in the organoid and original tissue is shown in Figure 5C; both indicate overexpression of T > C/A > G and C > T/G > A transversions, followed by C > G/G > C and C > A/G > T. In terms of the global variant profile, the IHCC organoid was found to retain appropriately 87% of the variants as that of the original tissue. In detail, 89% of the single nucleotide variants (shown in Figure 5D) and 82% of indels (not shown) in the original tissue were well retained in the IHCC organoid. Additionally, some representative genetic alterations can be seen in Figure 5E. Both organoid and original tissue harbored the MET, ARID1A, MUC5B, SMAD7, and IHD1 missense variants, an MSH3 non-frameshift insertion, and a MAP3K1 non-frameshift deletion.

### 3.4. Drug Screening

**Figure 6:**
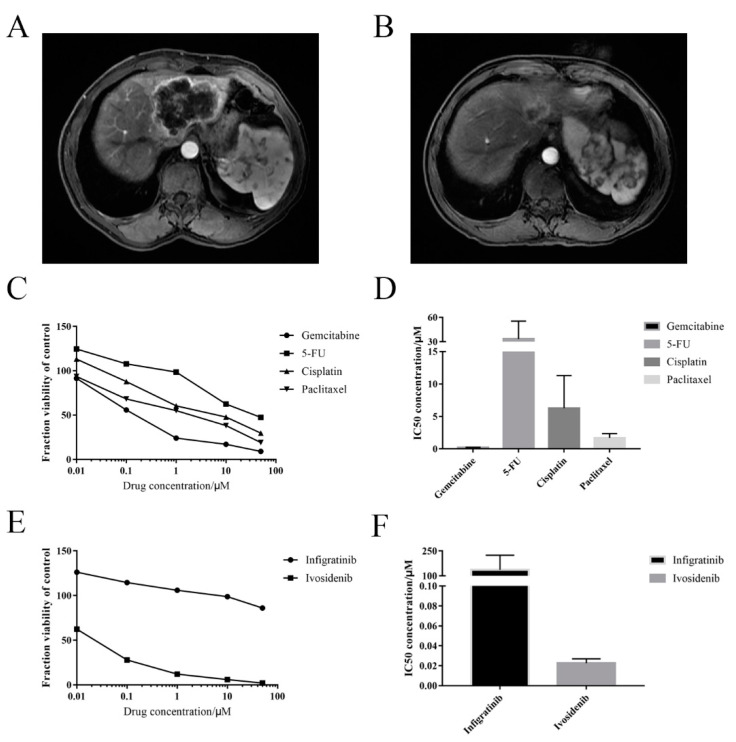
Drug screening of cancer organoid and validation of clinical case. (**A**,**B**) Magnetic resonance images of patient 8 at admission (**A**) and after receiving treatment of gemcitabine plus albumin-bound paclitaxel for three months (**B**). (**C**–**F**) Dose-response curves and half-maximal inhibitory concentrations (IC50) of the derived IHCC organoid treated with conventional chemotherapeutic drugs (**C**,**D**) and targeted drugs (**E**,**F**). Screening of each drug was performed in triplicate.

The established organoid was used for drug screening and tested with the chemotherapeutic agents, gemcitabine, 5-FU, cisplatin, paclitaxel; and the drugs used for targeted therapies, namely, infigratinib and ivosidenib. Patient 8 received gemcitabine plus albumin-bound paclitaxel treatment as conversion therapy for three months. Magnetic resonance images (Figure 6A,B) indicated partial response of the tumor after treatment. These findings were consistent with the results of drug screening using the cancer organoid (Figure 6C). Among the four chemotherapeutic drugs that were tested, gemcitabine and paclitaxel exhibited the strongest inhibitory effects on the cancer organoids, with half-maximal inhibitory concentrations (IC50) of 0.1965 μM and 1.697 μM, respectively (Figure 6D). Moreover, the inhibitor of the isocitrate dehydrogenase 1 (IDH1) family, ivosidenib, exhibited a strong inhibitory effect on the IHCC organoid, with an IC50 of 0.0226 μM (Figure 6E,F). This could likely be attributed to the IDH1 missense variant as seen in Figure 5E. On the other hand, the fibroblast growth factor receptor (FGFR) inhibitor, infigratinib, exhibited a marginal inhibitory effect on the cancer organoid.

## 4. Discussion

In this study, we evaluated the present situation of IHCC conversion therapy in a single study center. The results confirmed the effectiveness of conversion therapy using IHCC; however, the success rate of downstaging using conversion therapy in patients was relatively low (21.4%), and thereby, warranted an in vitro cell model to direct the treatment pattern. We established a cancer organoid of IHCC and determined that it could mimic the structural characteristics and retain the gene expression profiles of the original IHCC tissues. Moreover, the results obtained after drug screening using the cancer organoid was consistent with the clinical responses of patients, indicating the ability and effectiveness of the organoid to design a treatment pattern for conversion therapy.

Advanced IHCC was previously considered unsuitable for surgical treatment; however, conversion therapy improved the prognosis of patients to some extent. As reported in a systematic review, 27 of the 132 patients across 10 studies were downstaged successfully using conversion therapy and 23 patients were reported to be alive at the last follow-up that was conducted [25]. The survival rate of downstaged patients in the systematic review was 85.2% and was similar to that obtained in our study (77.8%). A study by Edeline J. et al. reports that 9 patients were downstaged successfully using radioembolization plus chemotherapy, of which 8 patients survived after 24 months of follow-up [18]. Moreover, Riby D. et al. compared the overall survival of 137 initially resectable patients and 32 successfully downstaged patients, showing no significant difference between them [26]. However, the response rate of conversion therapy is still low. The overall response rate has been reported as 17.3% in the systematic review and was found to be 21.4% in our study [25]. The main reason why downstaging failed in 33 of included 42 patients is that tumors in these 33 patients were resistant to received drugs or local treatment. On the other hand, Le Roy B. et al. reported a much higher response rate of 53% in locally advanced IHCC patients due to a rather high sensitivity of enrolled IHCC to conversion therapy [27]. The most effective treatment pattern was found to be transarterial radioembolization alone, while chemotherapy with or without radioembolization was the most commonly used therapy in our study. The selection of different conversion strategies was based on the suggestions of guidelines combined with personal experience. An improvement in drug selection for chemotherapy would be the key to increase the response rate of conversion therapy and prolong the overall survival of patients with IHCC.

The concept of using cancer organoids was proposed several decades ago and has been used as a model for drug screening in recent years. Ganesh K. et al. found that the drug sensitivity of the cancer organoid-based platform is positively correlated with clinical response [21]. Some other studies have also established the effectiveness of cancer organoids in drug screening. However, a majority of the studies did not correlate the results from drug screening with clinical response [24,28,29], because surgically resected cancer tissues were used as original specimens of the cancer organoids. It is known that the postoperative recurrence of the tumor and the prognosis of patients does not depend solely on the response of the tumor to chemotherapeutic drugs, but is also attributed to the negative margin, lymph node metastases, and the extent of vascular invasion. Based on these factors, the drug screening of cancer organoids derived from surgically resected tissues often poses a challenge to be correlated with the clinical responses in patients. In this study, we used specimens from successfully downstaged patients with IHCC for organoid establishment within 3 weeks, in whom the drug sensitivity to the tumor was confirmed. As expected, gemcitabine and paclitaxel were found to be the most effective drugs in the cancer organoid model, whereas gemcitabine plus albumin-bound paclitaxel induced a partial response in the patient.

A more suitable approach could likely entail the collection of tumor tissues using needle biopsies for organoid establishment in patients with initially unresectable cancer, followed by comparison with the clinical responses obtained based on drug screening. Nuciforo S. et al. attempted this approach in liver cancers and obtained hepatocellular carcinoma organoids and IHCC organoids at relatively low success rates of 33.3% and 60%, respectively [30]. In this study, our attempts at establishing an organoid using needle biopsy tissues proved to be unsuccessful. This procedure requires a precise puncture depth at a specific site containing an adequate amount of cancerous tissue. IHCC tissues containing extensive stromal cells may lead to multiple punctures and pose a high risk of bleeding. Therefore, the method to successfully obtain specimens to establish cancer organoids needs improvement.

Targeted therapy for IHCC has been explored for several decades. FGFR and IDH1 inhibitors are the two most studied class of compounds in recent times. FGFR variants are often observed in several cancer types including IHCC. FGFR-containing fusions caused by chromosomal translocations have been detected in 10–15% of the cases of IHCC and are known to play a vital role in the proliferation and metastasis of IHCC [31,32]. It has been reported that treatment with the FGFR inhibitor, infigratinib, may be effective in patients with FGFR-driven HCC [33]. Unfortunately, the IHCC samples in this study were not found to harbor the FGFR variant, and infigratinib did not exert a significant effect on the cancer organoid in vitro. On the contrary, IHD1, another common variant gene in IHCC accounting for up to 25% of cases [34], was detected in IHCC tissues and the derived organoids in our study. Accordingly, the IDH1 inhibitor, ivosidenib, showed remarkable inhibitory effects on the cancer organoid in vitro. Results of a phase 1 study suggest that ivosidenib is a well-tolerated option in patients with IDH1-variant cholangiocarcinoma [35], and phase 3 study shows progression-free survival was significantly improved with ivosidenib compared with placebo in advanced, IDH1-mutant cholangiocarcinoma [36]. In our study, we found that the organoids could be helpful in providing directions for targeted therapy by retaining some key genetic variants of the original tissue.

Our study had several limitations. First, it comprised only a small sample size of 42 patients receiving conversion therapy and the period of follow up was relatively short, which may lead to bias in data analysis. Moreover, many IHCC patients admitted to our hospital were diagnosed as metastatic stage and refused any treatment due to financial pressure or other reasons, which may result in some referral bias. A multi-center study would have been a better choice considering the low incidence of IHCC. Secondly, the cancer organoid established in the current study was relatively small compared to that in our previous study, which posed several challenges while performing immunohistochemistry and immunofluorescence analyses [23,24]. Therefore, we further used WES to compare the gene expression profiles of the organoid and the original tissue. Thirdly, the organoid in this study was established based on a conventional procedure [37]. Such organoids cannot be used for drug screening from the point of immunotherapy owing to a lack of immune cells. Novel methods to establish cancer organoids and reserve stromal cells have been recently proposed, which may provide a feasible approach to investigate the effects of immunotherapy in cancer [38].

## 5. Conclusions

To conclude, conversion therapy, as a novel treatment modality in IHCC, can result in an improvement in overall survival and better prognosis of patients. The low success rate of downstaging in conversion therapy continues to pose a challenge. The therapeutic approach using cancer organoids could likely play a vital role in directing conversion therapy owing to its ability to retain the original genetic expression profile and drug sensitivity.

## Figures and Tables

**Table 1:** Demographic and clinical data of patients receiving conversion therapy.

Variables	Successfully Downstaged Patients	Unsuccessfully Downstaged Patients	*p* Value
Number	9	33	
Age, years	64 (61–71)	62 (52.5–68.5)	0.450
Gender			0.784
Male	5 (55.6%)	20 (60.6%)	
Female	4 (44.4%)	13 (39.4%)	
Liver cirrhosis	1 (11.1%)	5 (15.2%)	0.759
Multiple lesions (≥2)	2 (22.2%)	20 (60.6%)	0.062
Tumor size			0.655
<5cm	1 (11.1%)	8 (24.2%)	
≥5cm	8 (88.9%)	25 (75.8%)	
Vascular invasion	3 (33.3%)	20 (60.6%)	0.257
Perineural invasion	1 (11.1%)	0 (0%)	0.214
Histological grade			0.004
Well	0 (0%)	1 (3.0%)	
Moderate	1 (11.1%)	0 (0%)	
Poor	8 (88.9%)	12 (36.4%)	
Unknown	0 (0%)	20 (60.6%)	
AJCC T stage			0.008
T1a	1 (11.1%)	2 (6.1%)	
T1b	3 (33.3%)	0 (0%)	
T2	2 (22.2%)	23 (69.7%)	
T3	1 (11.1%)	5 (15.2%)	
T4	2 (22.2%)	5 (15.2%)	
AJCC N stage			0.593
N0	3 (33.3%)	4 (12.1%)	
N1	6 (66.7%)	29 (87.9%)	
AJCC M stage			1.000
M0	9 (100%)	33 (100%)	
M1	0 (0%)	0 (0%)	

AJCC: American Joint Committee on Cancer.

**Table 2:** Detailed characteristics of successfully downstaged patients by conversion therapy.

Patient No.	Age	Gender	Tumor Size (cm)	Histological Grade	Tnm Stage	Treatment Pattern	Radial Margin Status	Recurrence Time (Months)	Status	Survival Time (Months)
Patient 1	73	Female	5.8	Poor	T1bN0M0	TACE	R0	No	Alive	84
Patient 2	44	Male	3	Poor	T1aN1M0	Gemcitabine	R0	No	Alive	8
Patient 3	72	Male	8.9	Poor	T3N1M0	Gemcitabine+Oxaliplatin	R0	29	Dead	39
Patient 4	62	Female	6	Moderate	T4N0M0	Radiotherapy	R0	21	Alive	21
Patient 5	64	Female	6.6	Poor	T1bN1M0	TACE	R0	No	Alive	30
Patient 6	70	Female	5	Poor	T1bN1M0	Gemcitabine+Oxaliplatin+Toripalimab	R0	No	Alive	8
Patient 7	67	Male	5.5	Poor	T2N0M0	Gemcitabine+Cisplatin	R0	6	Alive	11
Patient 8	62	Male	8.6	Poor	T2N1M0	Gemcitabine+albumin-bound paclitaxel	R0	No	Alive	5
Patient 9	60	Male	9.2	Poor	T4N1M0	Gemcitabine+albumin-bound paclitaxel	R0	No	Dead	3

TACE: transcatheter arterial chemoembolization.

## Data Availability

The data presented in this study are available on request from the corresponding author. The data are not publicly available due to the privacy of enrolled patient.
